# Inflammation-sleep interface in brain disease: TNF, insulin, orexin

**DOI:** 10.1186/1742-2094-11-51

**Published:** 2014-03-21

**Authors:** Ian A Clark, Bryce Vissel

**Affiliations:** 1 Biomedical Sciences and Biochemistry, Research School of Biology, Australian National University, Acton, Canberra, Australian Capital Territory 0200, Australia; 2 Neurodegeneration Research Group, Garvan Institute, 384 Victoria Street, Sydney, New South Wales 2010, Australia

**Keywords:** Alzheimer’s disease, coma, encephalopathy, IL-1, orexin, Parkinson’s disease, sleep, stroke, TNF, traumatic brain injury

## Abstract

The depth, pattern, timing and duration of unconsciousness, including sleep, vary greatly in inflammatory disease, and are regarded as reliable indicators of disease severity. Similarly, these indicators are applicable to the encephalopathies of sepsis, malaria, and trypanosomiasis, and to viral diseases such as influenza and AIDS. They are also applicable to sterile neuroinflammatory states, including Alzheimer’s disease, Parkinson’s disease, traumatic brain injury, stroke and type-2 diabetes, as well as in iatrogenic brain states following brain irradiation and chemotherapy. Here we make the case that the cycles of unconsciousness that constitute normal sleep, as well as its aberrations, which range from sickness behavior through daytime sleepiness to the coma of inflammatory disease states, have common origins that involve increased inflammatory cytokines and consequent insulin resistance and loss of appetite due to reduction in orexigenic activity. Orexin reduction has broad implications, which are as yet little appreciated in the chronic inflammatory conditions listed, whether they be infectious or sterile in origin. Not only is reduction in orexin levels characterized by loss of appetite, it is associated with inappropriate and excessive sleep and, when dramatic and chronic, leads to coma. Moreover, such reduction is associated with impaired cognition and a reduction in motor control. We propose that advanced understanding and appreciation of the importance of orexin as a key regulator of pathways involved in the maintenance of normal appetite, sleep patterns, cognition, and motor control may afford novel treatment opportunities.

## Introduction: TNF and IL-1 in disease pathogenesis

A number of proinflammatory (and indeed anti-inflammatory) cytokines exist, but for conciseness, comments will largely be restricted to tumor necrosis factor (TNF) and interleukin-1 (IL-1). In practice, this will mean IL-1β, since its twin, IL-1α, mostly avoids assay by remaining cell-bound, and is thus absent from serum. In brief, TNF activates NLRP3, a NOD-like receptor, which in turn activates caspase-1, which, as the IL-1β cleavage enzyme, converts the TNF-induced precursor, pro-IL-1β, to active IL-1β [1]. Studies in a rheumatoid arthritis context make the case for TNF being the master cytokine that initiates the inflammatory cascade [2]. In addition, being the specific target of a number of biological drugs in wide use gives TNF high profile in the disease literature. TNF and IL-1 share many functions [3], including the capacity to induce each other and interleukin-6 (IL-6) [4]. This cytokine has a number of important activities, and is often a convenient marker for inflammatory reactions because it appears in the circulation later, when illness is more evident, and it remains at higher levels for longer than either IL-1 or TNF. Both TNF [5] and IL-1 [6] are phylogenetically ancient, as are orexin (hypocretin) [7] and insulin [8], two mediators discussed here because of their functional alliance to TNF and IL-1, arguably present for many millions of years.

Both TNF and IL-1 have proved to be both ubiquitous and pleiotropic, and if one is present, the other typically will be also. While often grouped on their capacity to mediate innate immunity, they have physiological and disease roles that, at least in the literature, overshadow their immune functions. Hence, while often termed proinflammatory cytokines, in increasing concentrations they modulate normal physiology (including physiological sleep), the innate arm of the immune system, and inflammatory disease processes and progression. This occurs in conditions caused by infectious agents in general [9], and, as well as those discussed in this text, Crohn’s diseases [10], psoriasis [11], spondyloarthritis [12], rheumatoid arthritis [13], amyotrophic lateral sclerosis [14], Behçet’s disease [15], graft-versus-host disease [16], acute heart failure [17], preeclampsia [18], and autoimmunity in general, as well as aspects of the illness that accompanies malignancies [19].

## Inflammatory cytokines and sleep

Interferon was one of the first (1983) cytokines to be implicated in sleep [20] but has been less investigated than others, probably because its species specificity limits generalizations. The following year IL-1, previously known as endogenous pyrogen (the link being unexpectedly made because both were identical to serum amyloid A-inducer and lymphocyte-activating factor (LAF) [21]) was first associated with sleep [22]. TNF, first described in 1975 for its *in vivo* capacity to kill tumor cells [23], was, six years later, shown to kill malaria parasites *in vivo*, and proposed, along with IL-1 (then known as LAF), to cause the disease complexities of malaria and sepsis [24,25]. While sleep aberrations are part of these conditions, they were not singled out as a particular outcome of the presence of these cytokines. Soon after becoming available in recombinant form, these cytokines were confirmed to be linked to physiological sleep in 1987 [26], and an awareness developed of the metabolic and disease relevance of this association [27]. As reviewed in 1995 [28], this group and others had, by then, done considerable work on these effects being amplified by the increased cytokines generated by microbial infections, and also the implications of their functional redundancy. Moreover, just before the normal time of sleep onset for rats, TNF levels in brain tissue were shown to be 10-fold higher than their daily minimum [29]. Diurnal variations of the soluble TNF receptors (two forms exist, induced by increases in TNF) in plasma from healthy human volunteers are consistent with this model [30]. Key steps in establishing the importance of TNF in sleep were its suppression by an anti-TNF antibody [31] and both spontaneous and influenza-induced sleep being variously altered in double TNF receptor-deficient mice [32]. In brief, when uninfected, these mice had less non-rapid eye movement sleep (NREMS) than wild-type mice at night-time and more rapid eye movement sleep (REMS) than control mice during the day, whereas challenge with mouse-adapted influenza X-31 enhanced NREMS and decreased REMS in both strains to roughly the same extent. In addition, the strain lacking TNF receptors had higher levels of orexin mRNA. As recently summarized [33], wakefulness enhances TNF protein levels and expression in brain, and the highest normal brain levels, at least in the rat, occur at the time of usual sleep onset. Sleep deprivation elevates levels even further, the effects of which we experience in jetlag.

The nocturnal surge of melatonin that arises in the pineal gland, and determines the synchronization of pineal function with the diurnal cycle, has been studied extensively in normal physiology. Melatonin is, however, relatively absent from the literature on sleep variation in disease, with the exception of a recent valuable contribution [34]. In brief, therefore, we note that melatonin is well-recognized as an inhibitor of TNF [35-37], and that TNF, in turn, transiently inhibits its production [38]. Inferences regarding the previous paragraph can be drawn from these observations.

## The roles of orexin (hypocretin), including sleep/wake cycles

Orexin, a pleiotropic neuropeptide recently reviewed in detail [39], is a member of the incretin gene family of peptides [40,41], to which glucagon-like peptide-1 (GLP-1), discussed later, belongs. In brief, orexin has two isoforms, orexin A (hypocretin-1) and orexin-B (hypocretin-2), a single precursor protein, and two ubiquitously distributed receptors (OXR1 and OXR2), details of which need not concern a brief overview such as this. As recently reviewed [42], neurons that synthesize orexin are located in the lateral hypothalamus, said to be the key executive function site in the central nervous system. For decades, it has been well documented that this site governs core survival behaviors, such as sleep/wake cycles, energy metabolism, fight, flight, and food consumption. Typically orexin reaches critical sites in the brain through elaborate innervation throughout the brain, particularly in regions related to wakefulness [43]. Evidence for cerebrospinal fluid (CSF) levels of orexin reflecting its degree of neurotransmission, or even functional meaning, is argued to be still lacking [44]. Indeed, it seems safest to speak of this neuropeptide in terms of the degree of activity in the hypothalamic orexin neuronal network [45]. When levels of this activity are high, it orchestrates the appropriate levels of alertness required for planning and executing goal-oriented behaviors [45]. Low levels of orexin initiate sleep, and very low levels coma. Although still off the beaten path of many medical researchers, this neuropeptide may, in addition to its many other roles, be as close as we have yet come to understanding what modulates sleep depth and the sleep/wake cycle [46]. The involvement of orexin in the sleep pathology of neuroinflammatory diseases is discussed later.

## The concept of sleep rinsing the brain of molecules that accumulate while awake

In the absence of a lymphatic circulation to remove excess interstitial protein, the brain relies on its interstitial spaces, and thence the CSF, to serve this purpose. A recent report of a dramatic and quite unexpected diurnally cyclic event may well have rewritten assumptions of extracellular fluid flows in the mouse brain [47,48], and thus paved the way for novel explanations of sleep and related phenomena in mammals in general. Briefly, influx into the brain interstitial space of a tracer introduced into the CSF was reduced by ~95% in awake as compared with sleeping mice, arguing that the space to which CSF has access is considerably enlarged during sleep. In other words, the flow of CSF through the interstitial space is reduced during waking to only 5% of the flow found in sleep. Since an author of this work had earlier shown that interstitial fluid levels of amyloid-β (Aβ) in the brains of amyloid precursor protein (APP) transgenic mice correlated with time spent awake, and were significantly increased by chronic sleep restriction [49], radiolabeled soluble Aβ clearance was monitored, and levels were found to fall at twice the rate in sleeping than waking mice. It seems reasonable to predict that other molecules used as markers of Alzheimer’s disease (AD), such as pTau, α-synuclein and TNF, will prove to clear at the same rate as Aβ during sleep, with practical implications for timing CSF collection when studying patients.

## Implication for interpreting recently published data

Although these observations in mice are yet to be applied to studies of the human brain, or duplicated independently, their capacity to allow alternative interpretations of data is already impressive. For example, infusing the dual orexin receptor antagonist, almorexant, used to treat insomnia, into the cerebral ventricles suppresses the level of Aβ in brain interstitial fluid, and abolishes the natural diurnal variation of Aβ [49]. Moreover, systemic treatment with almorexant once daily for 8 weeks decreased Aβ plaque formation in the brain of APP transgenic mice [49,50]. However, almorexant would have considerably increased sleep time, so the period of brain flushing would increase considerably. One can therefore predict that Aβ, or any other free molecule in the brain interstitial fluid, would, purely by fluid mechanics, have little opportunity to accumulate post-almorexant. Nor would it show a diurnal pattern.

## Implications for normal diurnal changes in brain inflammatory cytokines, and thus Aβ

The passive removal of either TNF or Aβ from the brain interstitial fluid is simply a case of going with the flow, since any protein in the cerebral interstitial space can be expected to be flushed away with the same kinetics as shown for Aβ [48]. Presumably, this regular diurnal removal of TNF would allow the activity of orexigenic neurons in the lateral hypothalamus to rise each morning, gearing up the individual to face the challenges of the day [51]. The more profound question is why, as each awake period progresses, the rise in TNF [33] and Aβ [49] in CSF should occur. Increases in inflammatory cytokines have recently been argued to arise from physiological neuronal activity orchestrating actions of immune cells, vascular cells and neurons [52]. The physiological rise of soluble Aβ in awake subjects can be expected to follow, and be a consequence of, the increase in levels of inflammatory cytokines in the CSF of the human volunteers referred to previously [49], since APP expression [53-55] and its cleavage to Aβ [56-59] require increases in these mediators. These data also explain raised levels of Aβ and AβPP proteins in infectious diseases [60-62], since pathogens stimulate TNF generation [9].

## Implications for the poor cognition of disturbed and limited sleep

Common experience shows us that chronically broken or lost sleep has a great cognitive cost, and the link is well documented [63]. Hospital admittance for major surgery illustrates the phenomenon, and procedures such as coronary artery bypass surgery provide an example. They tend to be followed by cognitive decline, and excessive cerebral levels of inflammatory cytokines have been implicated [64], with TNF particularly in the spotlight [65]. Current ideas on how such cytokines increase so dramatically in these patients include volatile anaesthetics [66] and mitochondrial DNA, which, like bacterial DNA is hypomethylated, released from cells disrupted by surgical trauma [67]. An additional contributor to this cytokine increase is likely to be short and fragmented sleep, a well-recognized hazard for hospital patients, especially those undergoing intensive care [68,69]. The novel data on diurnal changes in brain interstitial space discussed previously [48] predicts that absence, during intensive care, of the normal nocturnal cerebral rinse provided by a good night’s sleep will cause levels of brain TNF, already excessive, to accumulate further, worsening surgery-induced cognitive defects. As recently reviewed [70], the negative effects of sleep deprivation, and the associated effect of increased levels of TNF on learning and memory, synaptic plasticity and expression of cognition-related signaling molecules are active topics of research. A recent study of a wide array of inflammatory markers in healthy young adult volunteers who underwent 40 hours of total sleep deprivation demonstrates the principle [71].

## Sickness behavior, daytime sleepiness, and insulin resistance

Excessive generation of TNF and IL-1 in infectious and autoimmune diseases is associated with fever, fatigue, inanition, skeletal muscle catabolism, and a tendency to sleep during normal periods of wakefulness, a syndrome referred to as sickness behavior [72,73]. As has been noted [74], this syndrome appears to be the expression of a central motivational state that reorganizes the organism’s priorities to cope with the harmful effects of pathogens. This includes changes in the diurnal pattern, the mechanism for which has been shown to be suppressed expression of the PAR bZip clock-controlled genes *Dbp*, *Tef*, and *Hlf* and of the period genes *Per1*, *Per2*, and *Per3* by increased levels of TNF and IL-1, the two most-studied inflammatory cytokines [75]. These authors also reported that increased TNF interferes with the expression of *Dbp* in the suprachiasmatic nucleus and causes prolonged rest periods in the dark, the time when mice normally show spontaneous locomotor activity. Not surprisingly, therefore, elements of sickness behavior characterize all chronic inflammatory diseases, whether or not a pathogen has initiated the event. Should the reorganization of the animal’s resources overcome the pathogen or injury, and homeostasis be re-established, all is well. Should, however, the chronic inflammatory response be relentless and the reorganized metabolism and altered diurnal pattern continue unabated, it becomes a liability, potentially leading to a fatal outcome characterized by energy shutdown and anorexia [76]. More acute outcomes have additional distinctive clinical characteristics that have been argued to operate through the same principles [77].

As might therefore be expected, daytime sleepiness is a common manifestation of a disrupted diurnal cycle, and a characteristic of the continuing chronic inflammatory states largely driven by these two cytokines. An example is AD, in which clock gene function, and hence the diurnal cycle, was shown to be distorted [78] some years before it was appreciated that TNF and IL-1 are not only central players in the pathogenesis of this condition but also regulators of clock genes themselves (see previous paragraph). It had already been reported that the duration of daytime sleep in AD correlated with the degree of functional impairment [79,80]. Other examples of daytime sleepiness in chronic inflammatory states are Parkinson’s disease (PD) [81,82], traumatic brain injury (TBI) [83,84], stroke [85,86], heart failure [87,88], and type-2 diabetes (T2DM) [89].

Clock genes, present in all tissues, are closely orchestrated to maintain normal physiology and diurnal patterns [90]. They undergo insulin-dependent regulation [91]. Circadian clock oscillation is altered in the hearts and livers of mice in which diabetes has been generated with streptozotocin [92], and can be corrected by injecting insulin to overcome insulin resistance. This is consistent with GLP-1 mimetics being therapeutically useful against T2DM through their ability to correct insulin resistance [93], which is evidently present in sickness behavior [94,95]. One such agent in regular clinical use, exenatide, has been reported to shorten daytime sleepiness in patients with T2DM [96]. Conceivably this class of agents, being related to orexin (that is, hypocretin), through the incretin family, as mentioned, could also prove, through an ability to correct altered diurnal patterns, to improve daytime sleepiness in the range of conditions discussed in the previous paragraph. As we have recently reviewed [97], GLP-1 mimetics routinely prescribed for T2DM have been reported to improve experimental models of AD (reversed memory impairment and synaptic loss) [98], PD (preserved dopaminergic neurons) [99], TBI (reversed behavioral impairment and memory deficits) [100,101], and stroke (reduced brain damage and improved functional outcome) [99,102].

## Orexin in the sleep pathology of inflammatory brain diseases

Orexin neuron activity is suppressed by bacterial lipopolysaccharide (LPS), a cytokine inducer commonly used to model inflammatory disease, including abnormal sleepiness and anorexia [103-105]. It is also suppressed by TNF (for which LPS is the prototype inducer [23]) predominantly through this cytokine degrading the mRNA of orexin precursor in a time- and dose-dependent manner [106]. One might therefore predict that orexin activity is reduced in states in which consciousness is depressed and TNF is increased, such as TBI, septic encephalopathy, and the post-chemotherapy brain. All three of these conditions have been tested, and shown promise. For example in 44 consecutive TBI patients CSF orexin levels were abnormally low in 95% of moderately to severely affected individuals 1 to 4 days after trauma [107], and 6 months later levels were still significantly low in patients, with post-traumatic excessive daytime sleepiness [108]. Unfortunately, low orexin is yet to reach the review literature on high levels of TNF in TBI [109]. Mouse TBI data provide compatible orexin results [110], and in conjunction with an anti-TNF report in the same model [111], are ripe for TNF-orexin linkage. A series of reports [112] of TBI cases in which anti-TNF was administered, may then eventually lead to controlled human studies combining these same components.

In a similar vein, a mouse sepsis model has been used to demonstrate, histologically, a six-fold decrease in orexigenic activity in the hypothalamus 48 hours after cecal ligation and puncture [113]. Injecting 3 nmol orexin intracerebroventricular (i.c.v.), an amount and route previously shown to overturn narcolepsy in orexin-deficient mice, reversed all changes within an hour. Although this text did not focus on encephalopathy, it relates a transformation, caused by i.c.v. orexin, from lethargy and loss of response to several stimuli to agitation and hyper-responsiveness to the same stimuli. Likewise, poor sleep quality in patients after chemotherapy has been closely linked to their inflammatory markers [114]. In the post-chemotherapy brain, the pathogenesis of which involves excess TNF generation [115] and lowered orexigenic activity [116], i.c.v. orexin reversed fatigue (that is, restored voluntary ambulatory activity) in a mouse model [116].

In addition to that seen in sepsis, the encephalopathies of malaria (often referred to as cerebral malaria), trypanosomiasis, AIDS and influenza warrant examining to see if whether orexigenic neuronal activity is depleted, and i.c.v. orexin restores function, since deep prolonged pathological sleep (that is, reversible coma without rationale) and high TNF are already in place [117-122]. The orexin link has already been made with trypanosomiasis [123]. Regarding malaria, recent evidence that LPS suppresses orexigenic activity [105] is consistent with earlier arguments that LPS and malaria generate diseases that are fundamentally the same [124]. Subsequent reports of parallels between septic and malarial encephalopathies noted in immunohistological studies on patient material [125,126] strengthen the case further. The concept is also conceivable for post-radiotherapy brain, in which orexin levels have not been published, but fatigue is notable [127]. Side effects can be ameliorated when either an anti-TNF monoclonal antibody [128] or a GLP-1 mimetic [129], two agents expected to increase orexin output [106,130], is administered soon after irradiation in mouse models. It is also illuminating that the molecular response of the mouse brain within a few hours after low-dose irradiation down-regulates neural pathways associated with cognitive dysfunctions that are also reduced in AD [131]. A GLP-1 mimetic also ameliorates a mouse model of TBI [100,101], one of the high TNF conditions noted to exhibit reduced brain orexin [108,110].

The literature on orexin and both AD and PD, two conditions characterized by chronic inflammation and circadian alterations that include daytime sleepiness, has a complex history. Potentially, one side of this controversy places these diseases outside the logic arrived at for sepsis, TBI and chemotherapy brain, as discussed. This impression arises from the number of reports that orexin levels in CSF samples are not significantly different in clinical cases and controls in AD [132,133] or in PD [134,135]. The alternative arguments, in favor of directly examining the orexigenic activity in the hypothalamus, and of viewing CSF levels as being a diagnostic tool to confirm severe cases rather than useful for understanding pathogenesis of AD [136,137] and PD [44,138,139], are consistent with the reasoning and methods employed in the sepsis encephalopathy and chemotherapy literature cited previously. Since i.c.v. orexin is reported to restore function in these conditions [113,116], this second line of reasoning seems the most plausible. Given that high cerebral TNF is a common denominator in these conditions, it is an obvious next experimental step to see w this increase explains why hypothalamic orexigenic activity is reduced [106] in all the conditions in the previous few paragraphs. Certainly, clarified arguments on a possible key role of orexin in AD and PD would, for the reasons outlined, give additional weight to the relevance of anti-TNF agents and GLP-1 mimetics, in which there is already close interest, as rational treatments for these two conditions. It would also add further urgency to developing a specific orexin agonist.

## Orexin in cognition, appetite, and water intake

To understand the role of orexin deficiency in AD and PD it is also crucial to appreciate that this neuropeptide, which is depressed by TNF, performs a number of key roles in memory acquisition and consolidation [140,141], as well as in long-term potentiation [142-144]. These data are entirely consistent with anti-TNF and GLP-1 mimetics improving cognition, as recently reviewed [97]. Regarding the relative importance of inflammatory pathways (to which orexin belongs, since TNF suppresses it) and Aβ in AD, we note that orexin can improve memory, even in mice overproducing Aβ [145]. The poor appetite that is a component of sickness behavior and occurs in chronic inflammatory diseases, such as AD and PD [146,147], is also consistent [148,149] with orexin inhibition by TNF [106]. Likewise, i.c.v. orexin increases water intake [150], so a reduced physiological thirst response in AD [151] is not unexpected.

## Orexin in motor control

Several converging lines of evidence are consistent with orexin dependence of central motor control, including the stage being set by direct innervation from the orexigenic hypothalamic neurons to essential subcortical motor structures [152]. In addition, orexigenic neurons are increasingly active during movement [153,154], and injecting orexin into the midbrain triggers locomotion [155]. More recent work [156] has demonstrated that orexin (orexin A, acting via both receptors) enhances the sensitivity of neurons in the lateral vestibular nucleus. Thus, orexigenic activity, increased on demand, is reasoned [156] to regulate the muscle tone required for normal subtleties of vestibular-mediated posture, motor balance, and negative geotaxis. Clearly, these observations have implications for understanding aspects of neurodegenerative diseases in which chronic inflammation down-regulates orexin, as discussed.

## Therapeutic prospects and roles for orexin agonists

GLP-1 and exenatide, one of its two mimetics in clinical use, have been reported to excite orexin neurons in *ex vivo* hypothalamic slices [130]. If this translates to *in vivo*, these agents could be regarded as functionally similar to an orexin agonist. This rationalizes the capacity of GLP-1 mimetics to shorten daytime sleepiness in T2DM, as discussed earlier [96]. Moreover, insulin resistance occurs in orexin knockout mice [157], and hypothalamic orexin prevents insulin resistance in a stress model in mice [158]. Thus, excitation of orexin by exenatide [130] is an additional rationale for GLP-1 mimetics generating positive *in vivo* outcomes, beyond improving insulin resistance, in experimental models of AD [98] and PD [99], as well as T2DM. Since TNF inhibits orexin [106], orexin increase through exenatide [130] could be regarded as another anti-TNF effect of the GLP-1 mimetics, and is consistent with the literature on specific anti-TNF agents reducing pathological human sleep [159-162], as it does physiological sleep [31,163]. It also takes our understanding of exenatide shortening daytime sleepiness in T2DM patients [96] to another level.

Another therapeutic possibility for orexin excitation has arisen within the literature on administering the branched-chain amino acids (BCAAs), leucine, isoleucine, and valine. In brief, therapeutic interest in this trio began in the early 1970s when they were reported to reduce the muscle protein catabolism of chronic inflammation [164]. Therefore, BCAAs began to be investigated for possible utility to treat burns, sepsis, and trauma. Their popularity as an uncontrolled over-the-counter diet supplement at least minimizes toxicity concerns, as does a two-year trial in about 650 patients with liver cirrhosis [165]. As recently discussed [166], the scientific challenge has been to integrate various leads and identify a precise focus for BCAA research beyond making itself generally useful by generating more protein. A recent report of oral BCAAs activating orexigenic neurons and also ameliorating sleep fragmentation observed in TBI mice [167] may have provided such a focus. An improvement in power spectral density, which quantifies the strength of electroencephalography (EEG) signals, was also induced by this BCAA therapy. Previous data from this group on BCAAs improving an index of synaptic deficiency in TBI mice [168] is consistent with orexin being required for effective long-term potentiation, as discussed above [142-144].

Developing orexin antagonists to treat insomnia is an active research field [169]. Clearly, a pressing need exists for a specific orexin agonist, or mimetic, small enough to allow its subcutaneous injection because it passes the blood-brain barrier, allowing subcutaneous injection, as do the GLP-1 mimetics. Such a molecule has potential for treating the inflammatory brain states discussed previously, including TBI, AD, PD, and the encephalopathies of sepsis, AIDS, influenza and malaria, as well as narcolepsy and alcohol toxicity (see [170]). The principle has been demonstrated in very different systems that allow orexin to enter the CSF: intranasal orexin, alleviating cognitive deficits produced by loss of sleep in nonhuman primates [171] and human narcolepsy [172]; i.c.v. administration in an experimental model to treat the severe fatigue that can persist for months or years after chemotherapy [116], and, by the same route, administration to produce arousal effects on acute alcohol intoxication-induced coma in rats [170]. This is not unexpected, since orexin is associated with the regulation of stress, depression, and reward in alcohol dependence [173]. Hence, an orexin mimetic could be a useful addition to anti-TNF agents and GLP-1 mimetics for treating the excessive sleep and coma in inflammatory brain states, as well as their cognitive dimension. A recent orexin-replacing ‘designer drug’ provides a promising approach [174].

## Conclusions

**Figure 1:**
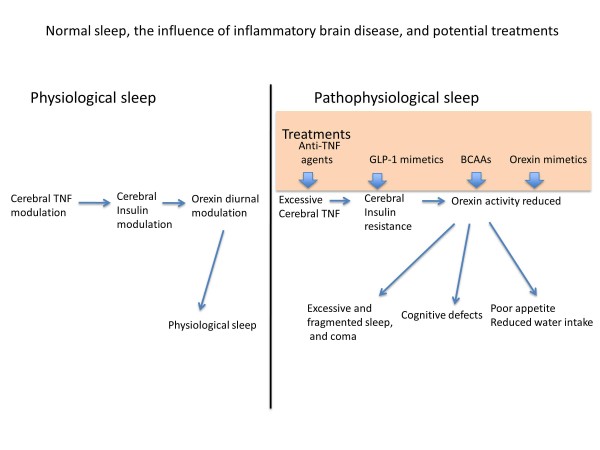
Normal sleep, the influence of inflammatory brain disease, and potential treatments.

This review argues the case that, as with other manifestations of inflammatory disease, pathological unconsciousness arises from distortions of the same cytokine and neuropeptide pathways that govern normal sleep. Specifically, the sleep aberrations seen in inflammatory illnesses, ranging from sickness behavior through daytime sleepiness to coma, have a common biological background involving increased inflammatory cytokines and consequent insulin resistance and orexin reduction. The logic of this literature reasons the relevance of anti-TNF agents and GLP-1 mimetics in treating these sleep aberrations, as well as the desirability of developing orexin mimetics for the purpose (Figure 1).

## Abbreviations

Aβ: amyloid-β; AD: Alzheimer’s disease; APP: amyloid precursor protein; BCAA: branch-chained amino acids; CSF: cerebrospinal fluid; EEG: electroencephalography; GLP-1: glucagon-like peptide-1; i.c.v.: intracerebroventricular; IL-1: interleukin-1; IL-6: interleukin-6; LAF: lymphocyte-activating factor; LPS: lipopolysaccharide; NLPR3: NOD-like receptor P3; NOD: nucleotide binding oligomerization domain; NREMS: non-rapid eye movement sleep; OXR1: orexin receptor 1; OXR2: orexin receptor 2; PD: Parkinson’s disease; REMS: rapid eye movement sleep; T2DM: type 2 diabetes mellitus; TBI: traumatic brain injury; TNF: tumor necrosis factor.

## Competing interests

The authors declare that they have no competing interests.

## Authors’ contributions

IAC proposed the scope of the review. Both authors were involved in planning and editing the manuscript, blending their complementary expertise. Both authors read and approved the final manuscript.
